# 6β-Acetamido-5α-hydroxy­cholestan-3β-yl acetate

**DOI:** 10.1107/S160053680803568X

**Published:** 2008-11-08

**Authors:** R. M.A. Pinto, M. Ramos Silva, A. Matos Beja, J. A. R. Salvador, J. A. Paixão

**Affiliations:** a Laboratório de Química Farmacêutica, Faculdade de Farmácia, Universidade de Coimbra, P-3000-295 Coimbra, Portugal; b CEMDRX, Departamento de Física, Faculdade de Ciências e Tecnologia, Universidade de Coimbra, P-3004-516 Coimbra, Portugal

## Abstract

The title steroid, C_31_H_53_NO_4_, was prepared from the corresponding 5α,6α-epoxy­cholestane. The conformation of the six-membered rings is close to a chair form, while the five-membered ring adopts a twist conformation. The hydroxyl and acetamide groups are in axial positions. The nucleophilic species bound to the steroid nucleus at position 6 by the β-face, whereas the hydroxyl group at position 5 has α-orientation. All rings are *trans*-fused. The crystal packing shows that the mol­ecules related by twofold symmetry exist as O—H⋯O hydrogen-bonded dimers.

## Related literature

For epoxy­steroid chemistry, see: Salvador *et al.* (2006 ▶, 2008 ▶); Pinto *et al.* (2008*a* ▶). For the synthesis of vicinal *N*-acyl hydroxy­amines, see: Pinto *et al.* (2006 ▶). For related steroid structures, see: Pinto *et al.* (2007*a* ▶,*b* ▶, 2008*b* ▶). For puckering parameters, see: Cremer & Pople (1975 ▶).
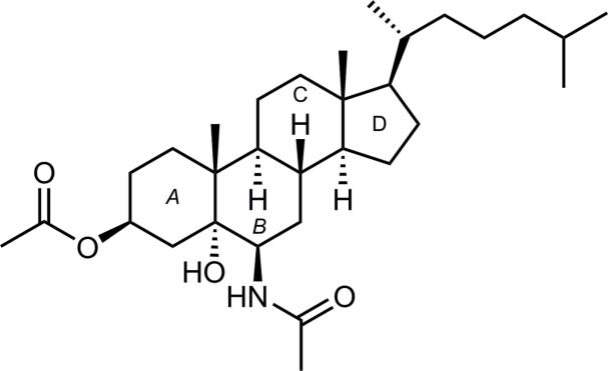


## Experimental

### 

#### Crystal data

C_31_H_53_NO_4_
*M* *_r_* = 503.74
Monoclinic, 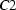

*a* = 31.4800 (12) Å
*b* = 10.0043 (4) Å
*c* = 9.7681 (4) Å
β = 94.276 (3)°
*V* = 3067.8 (2) Å^3^
*Z* = 4
Mo *K*α radiation
μ = 0.07 mm^−1^
*T* = 293 (2) K
0.40 × 0.20 × 0.14 mm

#### Data collection

Bruker APEXII CCD area-detector diffractometer
Absorption correction: multi-scan (*SADABS*; Sheldrick, 2000 ▶) *T*_min_ = 0.840, *T*_max_ = 0.990
43053 measured reflections
4691 independent reflections
2907 reflections with *I* > 2σ(*I*)
*R*_int_ = 0.045

#### Refinement

*R*[*F* ^2^ > 2σ(*F* ^2^)] = 0.050
*wR*(*F* ^2^) = 0.162
*S* = 1.03
4691 reflections
333 parameters
1 restraint
H-atom parameters constrained
Δρ_max_ = 0.34 e Å^−3^
Δρ_min_ = −0.24 e Å^−3^

### 

Data collection: *SMART* (Bruker, 2003 ▶); cell refinement: *SAINT* (Bruker, 2003 ▶); data reduction: *SAINT*; program(s) used to solve structure: *SHELXS97* (Sheldrick, 2008 ▶); program(s) used to refine structure: *SHELXL97* (Sheldrick, 2008 ▶); molecular graphics: *PLATON* (Spek, 2003 ▶); software used to prepare material for publication: *SHELXL97*.

## Supplementary Material

Crystal structure: contains datablocks global, I. DOI: 10.1107/S160053680803568X/ci2694sup1.cif

Structure factors: contains datablocks I. DOI: 10.1107/S160053680803568X/ci2694Isup2.hkl

Additional supplementary materials:  crystallographic information; 3D view; checkCIF report

## Figures and Tables

**Table 1:** Hydrogen-bond geometry (Å, °)

*D*—H⋯*A*	*D*—H	H⋯*A*	*D*⋯*A*	*D*—H⋯*A*
O5—H5*A*⋯O6^i^	0.82	1.99	2.804 (3)	172

Symmetry code: (i) 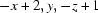.

**Fig. 1.**
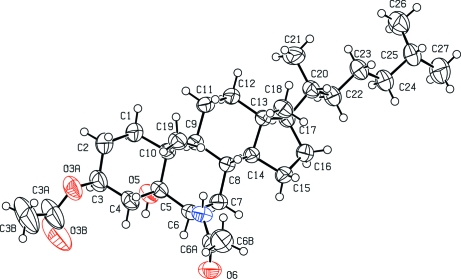
Molecular structure of the title compound. Displacement ellipsoids are drawn at the 50% probability level.

## supplementary crystallographic information

### Comment

Epoxysteroids are useful synthetic intermediates in the synthesis of important biologically active molecules (Salvador *et al.*, 2006, 2008; Pinto *et al.*, 2008*a*). In fact, it is the stereochemistry of the starting epoxide that rules its nucleophilic ring-opening. Recently, using epoxides as substrates, we described an efficient one-pot procedure for the synthesis of vicinal *N*-acyl hydroxyamines by a bismuth(III) salt-promoted reaction (Pinto *et al.*, 2006). Later, we reported the X-ray crystal structure of 5α-acetamido-6β-hydroxy-20-oxoandrostan-3β-yl acetate obtained by the above mentioned reaction (Pinto *et al.*, 2007*a*). Since the starting epoxide has a 5β,6β-conformation, the nucleophile attacked the steroid nucleus at C5 by the α-face. The title compound has been obtained from the corresponding 5α,6α-epoxycholestane derivative, under similar reaction conditions, in 90% yield (Pinto *et al.*, 2006). The present communication unequivocally demonstrated the *trans*-diaxial nature of the 5α,6α-epoxide ring-opening by the bismuth(III) salt-catalyzed Ritter reaction. Related X-ray diffraction studies on 5α-hydroxy-6β-substituted steroids have been recently published by our group (Pinto *et al.*, 2007*b*, 2008*b*).

The conformations of the six-membered rings are close to a chair form, as shown by the Cremer & Pople (1975) puckering parameters [ring A: Q = 0.580 (3) Å, θ = 4.7 (4)° and φ = 276 (4)°; ring B: Q = 0.554 (3) Å, θ = 4.4 (3)° and φ = 318 (4)°; ring C: Q = 0.583 (3) Å, θ = 1.3 (3)° and φ = 20 (7)°]. The D-ring has a twisted conformation around the C13—C14 bond with puckering parameters q_2_ = 0.459 (3)Å and φ_2_ = 192.9 (4)°. All rings of the molecule are *trans* fused. The acetoxy group at C3 is equatorial to the A ring, and both substituents at ring B are axial. The amide group adopts the usual *trans* conformation.

The molecules are hydrogen-bonded through the hydroxyl group at C5, acting as donor towards the carbonyl O atom of the amide moiety. Remarkably, the H atom attached to the amide N atom is not involved in any hydrogen bond. This may arise from steric hindrance caused by the C19-methyl group. The following short intramolecular distances are also spotted in the structure: H1B···O5 2.42 Å, H9···O5 2.42 Å and H6···O6 2.39 Å.

The anisotropic displacement tensor of the terminal atoms of the 3β-acetoxy group are strongly anisotropic, suggesting a large amplitude of vibration of these atoms perpendicular to the mean plane of this group.

### Experimental

The synthesis of 6β-acetamido-5α-hydroxycholestan-3β-yl acetate was efficiently accomplished by nucleophilic ring-opening of the corresponding 5α,6α-epoxycholestane catalyzed by BiBr_3_ in acetonitrile (Pinto *et al.*, 2006). The product of this reaction was isolated in 90% yield and identified as the title compound from IR, ^1^H and ^13^C NMR spectroscopy data (Pinto *et al.*, 2006). Recrystallization from acetonitrile at room temperature gave colourless single crystals suitable for X-ray diffraction analysis.

### Refinement

H atoms were fixed geometrically (O—H = 0.82 Å, N—H = 0.86 Å and C—H = 0.96–0.98 Å) and treated as riding with *U*_iso_(H) = 1.2*U*_eq_(C) and 1.5*U*_eq_(O and methyl C). In the absence of significant anomalous scattering, Friedel pairs were merged prior to the final refinement. Though the absolute configuration was not determined from the X-ray data but was known from the synthetic route.

### Crystal data

**Table:**

C_31_H_53_NO_4_	*F*_000_ = 1112
*M**_r_* = 503.74	*D*_x_ = 1.091 Mg m^−^^3^
Monoclinic, *C*2	Mo *K*α radiation λ = 0.71073 Å
Hall symbol: C 2y	Cell parameters from 5555 reflections
*a* = 31.4800 (12) Å	θ = 2.4–22.9º
*b* = 10.0043 (4) Å	µ = 0.07 mm^−^^1^
*c* = 9.7681 (4) Å	*T* = 293 (2) K
β = 94.276 (3)º	Truncated parallelipiped, clear colourless
*V* = 3067.8 (2) Å^3^	0.40 × 0.20 × 0.14 mm
*Z* = 4	

### Data collection

**Table:**

Bruker APEXII CCD area-detector diffractometer	4691 independent reflections
Radiation source: fine-focus sealed tube	2907 reflections with *I* > 2σ(*I*)
Monochromator: graphite	*R*_int_ = 0.045
*T* = 293(2) K	θ_max_ = 30.1º
φ and ω scans	θ_min_ = 1.3º
Absorption correction: multi-scan(SADABS; Sheldrick, 2000)	*h* = −43→44
*T*_min_ = 0.840, *T*_max_ = 0.990	*k* = −13→13
43053 measured reflections	*l* = −13→13

### Refinement

**Table:**

Refinement on *F*^2^	Secondary atom site location: difference Fourier map
Least-squares matrix: full	Hydrogen site location: inferred from neighbouring sites
*R*[*F*^2^ > 2σ(*F*^2^)] = 0.050	H-atom parameters constrained
*wR*(*F*^2^) = 0.162	*w* = 1/[σ^2^(*F*_o_^2^) + (0.0835*P*)^2^ + 0.6891*P*] where *P* = (*F*_o_^2^ + 2*F*_c_^2^)/3
*S* = 1.03	(Δ/σ)_max_ = 0.001
4691 reflections	Δρ_max_ = 0.34 e Å^−^^3^
333 parameters	Δρ_min_ = −0.24 e Å^−^^3^
1 restraint	Extinction correction: none
Primary atom site location: structure-invariant direct methods	

### Special details

**Table:**

Geometry. All e.s.d.'s (except the e.s.d. in the dihedral angle between two l.s. planes) are estimated using the full covariance matrix. The cell e.s.d.'s are taken into account individually in the estimation of e.s.d.'s in distances, angles and torsion angles; correlations between e.s.d.'s in cell parameters are only used when they are defined by crystal symmetry. An approximate (isotropic) treatment of cell e.s.d.'s is used for estimating e.s.d.'s involving l.s. planes.
Refinement. Refinement of *F*^2^ against ALL reflections. The weighted *R*-factor *wR* and goodness of fit *S* are based on *F*^2^, conventional *R*-factors *R* are based on *F*, with *F* set to zero for σ(*F*^2^) is used only for calculating *R*-factors(gt) *etc*. and is not relevant to the choice of reflections for refinement. *R*-factors based on *F*^2^ are statistically about twice as large as those based on *F*, and *R*- factors based on ALL data will be even larger.

### Fractional atomic coordinates and isotropic or equivalent isotropic displacement parameters (Å^2^)

**Table:**

	*x*	*y*	*z*	*U*_iso_*/*U*_eq_	
C1	0.87141 (10)	−0.0088 (3)	0.1952 (3)	0.0615 (8)	
H1A	0.8421	−0.0361	0.1741	0.074*	
H1B	0.8806	0.0391	0.1163	0.074*	
C2	0.89921 (11)	−0.1341 (3)	0.2196 (4)	0.0727 (9)	
H2A	0.8880	−0.1876	0.2916	0.087*	
H2B	0.8978	−0.1874	0.1365	0.087*	
C3	0.94513 (11)	−0.0993 (4)	0.2603 (4)	0.0678 (9)	
H3	0.9583	−0.0598	0.1820	0.081*	
C4	0.94903 (10)	−0.0042 (3)	0.3809 (3)	0.0592 (7)	
H4A	0.9786	0.0217	0.3991	0.071*	
H4B	0.9400	−0.0490	0.4618	0.071*	
C5	0.92162 (8)	0.1215 (3)	0.3521 (3)	0.0484 (6)	
C6	0.92943 (8)	0.2272 (3)	0.4659 (3)	0.0500 (6)	
H6	0.9595	0.2526	0.4664	0.060*	
C7	0.90374 (8)	0.3533 (3)	0.4310 (3)	0.0508 (6)	
H7A	0.9156	0.3976	0.3541	0.061*	
H7B	0.9066	0.4136	0.5089	0.061*	
C8	0.85643 (8)	0.3267 (3)	0.3944 (3)	0.0424 (5)	
H8	0.8434	0.2971	0.4773	0.051*	
C9	0.84930 (8)	0.2180 (3)	0.2834 (3)	0.0443 (5)	
H9	0.8610	0.2530	0.2005	0.053*	
C10	0.87356 (8)	0.0865 (3)	0.3205 (3)	0.0466 (6)	
C11	0.80110 (9)	0.1986 (3)	0.2483 (3)	0.0550 (7)	
H11A	0.7968	0.1321	0.1763	0.066*	
H11B	0.7882	0.1653	0.3287	0.066*	
C12	0.77895 (9)	0.3288 (3)	0.2008 (3)	0.0525 (7)	
H12A	0.7898	0.3571	0.1152	0.063*	
H12B	0.7487	0.3123	0.1836	0.063*	
C13	0.78570 (8)	0.4407 (3)	0.3063 (3)	0.0421 (6)	
C14	0.83406 (8)	0.4540 (3)	0.3415 (3)	0.0424 (5)	
H14	0.8462	0.4743	0.2543	0.051*	
C15	0.83868 (9)	0.5810 (3)	0.4253 (3)	0.0549 (7)	
H15A	0.8667	0.6201	0.4198	0.066*	
H15B	0.8340	0.5642	0.5209	0.066*	
C16	0.80370 (9)	0.6726 (3)	0.3580 (3)	0.0601 (7)	
H16A	0.8164	0.7448	0.3089	0.072*	
H16B	0.7869	0.7109	0.4274	0.072*	
C17	0.77525 (8)	0.5856 (3)	0.2575 (3)	0.0451 (6)	
H17	0.7864	0.5953	0.1670	0.054*	
C18	0.76160 (9)	0.4092 (3)	0.4336 (3)	0.0542 (7)	
H18A	0.7319	0.3972	0.4067	0.081*	
H18B	0.7650	0.4820	0.4976	0.081*	
H18C	0.7728	0.3289	0.4761	0.081*	
C19	0.85347 (10)	0.0158 (3)	0.4394 (3)	0.0579 (7)	
H19A	0.8272	−0.0254	0.4058	0.087*	
H19B	0.8480	0.0799	0.5090	0.087*	
H19C	0.8727	−0.0514	0.4775	0.087*	
C20	0.72882 (8)	0.6341 (3)	0.2425 (3)	0.0500 (6)	
H20	0.7178	0.6302	0.3337	0.060*	
C21	0.70059 (12)	0.5469 (4)	0.1483 (5)	0.0822 (12)	
H21A	0.7130	0.5372	0.0621	0.123*	
H21B	0.6730	0.5874	0.1335	0.123*	
H21C	0.6978	0.4605	0.1895	0.123*	
C22	0.72669 (9)	0.7808 (3)	0.1956 (3)	0.0543 (7)	
H22A	0.7450	0.8335	0.2591	0.065*	
H22B	0.7380	0.7869	0.1061	0.065*	
C23	0.68225 (10)	0.8419 (3)	0.1864 (4)	0.0604 (8)	
H23A	0.6656	0.8026	0.1090	0.072*	
H23B	0.6685	0.8193	0.2689	0.072*	
C24	0.68225 (10)	0.9932 (3)	0.1698 (3)	0.0602 (8)	
H24A	0.6956	1.0152	0.0862	0.072*	
H24B	0.6996	1.0318	0.2460	0.072*	
C25	0.63810 (11)	1.0583 (4)	0.1637 (4)	0.0655 (8)	
H25	0.6235	1.0251	0.2420	0.079*	
C26	0.61094 (14)	1.0219 (5)	0.0335 (4)	0.0889 (12)	
H26A	0.6241	1.0561	−0.0449	0.133*	
H26B	0.5831	1.0603	0.0369	0.133*	
H26C	0.6085	0.9265	0.0265	0.133*	
C27	0.64222 (15)	1.2093 (4)	0.1785 (5)	0.0938 (13)	
H27A	0.6586	1.2437	0.1073	0.141*	
H27B	0.6563	1.2304	0.2664	0.141*	
H27C	0.6144	1.2491	0.1710	0.141*	
O3A	0.96596 (8)	−0.2259 (3)	0.2984 (3)	0.0831 (7)	
C3A	1.00685 (17)	−0.2395 (6)	0.2845 (7)	0.118 (2)	
C3B	1.0213 (2)	−0.3779 (7)	0.3176 (9)	0.162 (3)	
H3BA	1.0490	−0.3754	0.3656	0.242*	
H3BB	1.0226	−0.4279	0.2341	0.242*	
H3BC	1.0015	−0.4199	0.3743	0.242*	
O3B	1.02871 (13)	−0.1488 (5)	0.2544 (7)	0.169 (2)	
O5	0.93451 (6)	0.1818 (2)	0.2284 (2)	0.0583 (5)	
H5A	0.9594	0.2062	0.2402	0.087*	
N6	0.92306 (7)	0.1766 (3)	0.6033 (2)	0.0574 (6)	
H6A	0.9009	0.1287	0.6138	0.069*	
O6	0.98188 (8)	0.2694 (4)	0.7050 (3)	0.0856 (8)	
C6A	0.94987 (10)	0.2006 (4)	0.7137 (3)	0.0608 (8)	
C6B	0.93864 (13)	0.1416 (6)	0.8461 (4)	0.0881 (12)	
H6BA	0.9595	0.0756	0.8757	0.132*	
H6BB	0.9111	0.1004	0.8339	0.132*	
H6BC	0.9381	0.2106	0.9142	0.132*	

### Atomic displacement parameters (Å^2^)

**Table:**

	*U*^11^	*U*^22^	*U*^33^	*U*^12^	*U*^13^	*U*^23^
C1	0.0589 (17)	0.0517 (17)	0.0728 (19)	0.0080 (14)	−0.0019 (14)	−0.0103 (15)
C2	0.070 (2)	0.0522 (18)	0.095 (2)	0.0131 (16)	0.0014 (17)	−0.0149 (17)
C3	0.0612 (18)	0.0521 (17)	0.091 (2)	0.0156 (15)	0.0111 (16)	0.0011 (16)
C4	0.0463 (15)	0.0544 (17)	0.077 (2)	0.0114 (13)	0.0071 (13)	0.0047 (15)
C5	0.0400 (13)	0.0490 (14)	0.0561 (15)	0.0049 (11)	0.0040 (11)	0.0057 (12)
C6	0.0358 (12)	0.0532 (16)	0.0604 (16)	−0.0013 (11)	0.0005 (11)	0.0039 (12)
C7	0.0416 (13)	0.0474 (15)	0.0627 (16)	−0.0027 (11)	−0.0015 (11)	−0.0031 (13)
C8	0.0371 (12)	0.0432 (13)	0.0467 (13)	−0.0005 (10)	0.0022 (10)	−0.0014 (10)
C9	0.0394 (12)	0.0420 (13)	0.0510 (14)	0.0014 (10)	0.0005 (10)	−0.0037 (10)
C10	0.0399 (13)	0.0411 (13)	0.0582 (15)	0.0009 (11)	0.0008 (11)	−0.0016 (11)
C11	0.0461 (14)	0.0497 (16)	0.0670 (17)	−0.0003 (12)	−0.0101 (12)	−0.0092 (13)
C12	0.0451 (14)	0.0510 (15)	0.0594 (16)	0.0052 (12)	−0.0083 (12)	−0.0098 (13)
C13	0.0389 (12)	0.0424 (13)	0.0450 (13)	−0.0002 (10)	0.0042 (10)	−0.0006 (10)
C14	0.0412 (13)	0.0404 (12)	0.0453 (13)	−0.0013 (10)	0.0022 (10)	−0.0006 (10)
C15	0.0482 (15)	0.0448 (14)	0.0704 (18)	0.0002 (12)	−0.0042 (13)	−0.0082 (13)
C16	0.0506 (15)	0.0471 (15)	0.082 (2)	0.0019 (13)	0.0019 (14)	−0.0093 (15)
C17	0.0444 (13)	0.0452 (13)	0.0462 (13)	0.0028 (11)	0.0056 (10)	0.0035 (11)
C18	0.0461 (14)	0.0585 (16)	0.0587 (16)	0.0008 (13)	0.0087 (12)	0.0103 (13)
C19	0.0488 (15)	0.0455 (15)	0.079 (2)	−0.0035 (12)	0.0051 (14)	0.0035 (14)
C20	0.0467 (14)	0.0486 (14)	0.0546 (15)	0.0056 (12)	0.0024 (11)	0.0031 (12)
C21	0.063 (2)	0.062 (2)	0.116 (3)	0.0088 (17)	−0.030 (2)	−0.008 (2)
C22	0.0556 (15)	0.0533 (16)	0.0545 (15)	0.0110 (13)	0.0067 (12)	0.0078 (13)
C23	0.0563 (17)	0.0579 (18)	0.0671 (18)	0.0147 (14)	0.0054 (13)	0.0103 (14)
C24	0.0612 (18)	0.0575 (18)	0.0628 (18)	0.0135 (14)	0.0099 (14)	0.0073 (14)
C25	0.0664 (19)	0.0594 (19)	0.0717 (19)	0.0188 (16)	0.0113 (16)	0.0060 (16)
C26	0.084 (3)	0.083 (3)	0.097 (3)	0.030 (2)	−0.008 (2)	0.001 (2)
C27	0.100 (3)	0.064 (2)	0.117 (3)	0.027 (2)	0.003 (2)	0.000 (2)
O3A	0.0696 (15)	0.0601 (14)	0.122 (2)	0.0235 (12)	0.0206 (13)	0.0056 (14)
C3A	0.088 (3)	0.082 (3)	0.191 (6)	0.038 (3)	0.054 (3)	0.027 (4)
C3B	0.134 (5)	0.105 (4)	0.253 (9)	0.077 (4)	0.064 (5)	0.042 (5)
O3B	0.091 (3)	0.117 (4)	0.308 (7)	0.035 (2)	0.077 (3)	0.051 (4)
O5	0.0489 (10)	0.0654 (13)	0.0616 (11)	0.0037 (10)	0.0108 (8)	0.0083 (11)
N6	0.0421 (12)	0.0699 (16)	0.0593 (13)	−0.0037 (12)	−0.0022 (10)	0.0048 (13)
O6	0.0545 (13)	0.125 (2)	0.0768 (15)	−0.0114 (15)	−0.0017 (11)	−0.0260 (16)
C6A	0.0482 (15)	0.072 (2)	0.0617 (17)	0.0159 (15)	−0.0013 (13)	−0.0125 (15)
C6B	0.084 (2)	0.115 (3)	0.064 (2)	0.013 (3)	0.0005 (17)	−0.002 (2)

### Geometric parameters (Å, °)

**Table:**

C1—C2	1.537 (4)	C17—C20	1.536 (4)
C1—C10	1.549 (4)	C17—H17	0.98
C1—H1A	0.97	C18—H18A	0.96
C1—H1B	0.97	C18—H18B	0.96
C2—C3	1.511 (5)	C18—H18C	0.96
C2—H2A	0.97	C19—H19A	0.96
C2—H2B	0.97	C19—H19B	0.96
C3—O3A	1.461 (4)	C19—H19C	0.96
C3—C4	1.512 (5)	C20—C21	1.509 (5)
C3—H3	0.98	C20—C22	1.537 (4)
C4—C5	1.539 (4)	C20—H20	0.98
C4—H4A	0.97	C21—H21A	0.96
C4—H4B	0.97	C21—H21B	0.96
C5—O5	1.436 (3)	C21—H21C	0.96
C5—C6	1.541 (4)	C22—C23	1.523 (4)
C5—C10	1.561 (4)	C22—H22A	0.97
C6—N6	1.461 (4)	C22—H22B	0.97
C6—C7	1.523 (4)	C23—C24	1.523 (5)
C6—H6	0.98	C23—H23A	0.97
C7—C8	1.528 (4)	C23—H23B	0.97
C7—H7A	0.97	C24—C25	1.532 (4)
C7—H7B	0.97	C24—H24A	0.97
C8—C14	1.527 (4)	C24—H24B	0.97
C8—C9	1.540 (3)	C25—C27	1.522 (6)
C8—H8	0.98	C25—C26	1.522 (6)
C9—C11	1.543 (4)	C25—H25	0.98
C9—C10	1.551 (4)	C26—H26A	0.96
C9—H9	0.98	C26—H26B	0.96
C10—C19	1.535 (4)	C26—H26C	0.96
C11—C12	1.532 (4)	C27—H27A	0.96
C11—H11A	0.97	C27—H27B	0.96
C11—H11B	0.97	C27—H27C	0.96
C12—C13	1.526 (4)	O3A—C3A	1.312 (5)
C12—H12A	0.97	C3A—O3B	1.190 (7)
C12—H12B	0.97	C3A—C3B	1.485 (7)
C13—C18	1.537 (4)	C3B—H3BA	0.96
C13—C14	1.541 (3)	C3B—H3BB	0.96
C13—C17	1.554 (4)	C3B—H3BC	0.96
C14—C15	1.513 (4)	O5—H5A	0.82
C14—H14	0.98	N6—C6A	1.341 (4)
C15—C16	1.542 (4)	N6—H6A	0.86
C15—H15A	0.97	O6—C6A	1.228 (4)
C15—H15B	0.97	C6A—C6B	1.488 (5)
C16—C17	1.547 (4)	C6B—H6BA	0.96
C16—H16A	0.97	C6B—H6BB	0.96
C16—H16B	0.97	C6B—H6BC	0.96
			
C2—C1—C10	112.9 (3)	C15—C16—H16B	110.2
C2—C1—H1A	109.0	C17—C16—H16B	110.2
C10—C1—H1A	109.0	H16A—C16—H16B	108.5
C2—C1—H1B	109.0	C20—C17—C16	112.6 (2)
C10—C1—H1B	109.0	C20—C17—C13	120.1 (2)
H1A—C1—H1B	107.8	C16—C17—C13	103.3 (2)
C3—C2—C1	112.1 (3)	C20—C17—H17	106.7
C3—C2—H2A	109.2	C16—C17—H17	106.7
C1—C2—H2A	109.2	C13—C17—H17	106.7
C3—C2—H2B	109.2	C13—C18—H18A	109.5
C1—C2—H2B	109.2	C13—C18—H18B	109.5
H2A—C2—H2B	107.9	H18A—C18—H18B	109.5
O3A—C3—C2	105.7 (3)	C13—C18—H18C	109.5
O3A—C3—C4	109.8 (3)	H18A—C18—H18C	109.5
C2—C3—C4	111.7 (3)	H18B—C18—H18C	109.5
O3A—C3—H3	109.9	C10—C19—H19A	109.5
C2—C3—H3	109.9	C10—C19—H19B	109.5
C4—C3—H3	109.9	H19A—C19—H19B	109.5
C3—C4—C5	111.1 (3)	C10—C19—H19C	109.5
C3—C4—H4A	109.4	H19A—C19—H19C	109.5
C5—C4—H4A	109.4	H19B—C19—H19C	109.5
C3—C4—H4B	109.4	C21—C20—C17	112.8 (3)
C5—C4—H4B	109.4	C21—C20—C22	111.1 (3)
H4A—C4—H4B	108.0	C17—C20—C22	110.5 (2)
O5—C5—C4	107.9 (2)	C21—C20—H20	107.4
O5—C5—C6	106.2 (2)	C17—C20—H20	107.4
C4—C5—C6	111.9 (2)	C22—C20—H20	107.4
O5—C5—C10	105.0 (2)	C20—C21—H21A	109.5
C4—C5—C10	112.0 (2)	C20—C21—H21B	109.5
C6—C5—C10	113.4 (2)	H21A—C21—H21B	109.5
N6—C6—C7	112.7 (2)	C20—C21—H21C	109.5
N6—C6—C5	113.5 (2)	H21A—C21—H21C	109.5
C7—C6—C5	110.7 (2)	H21B—C21—H21C	109.5
N6—C6—H6	106.5	C23—C22—C20	114.9 (3)
C7—C6—H6	106.5	C23—C22—H22A	108.6
C5—C6—H6	106.5	C20—C22—H22A	108.6
C6—C7—C8	113.6 (2)	C23—C22—H22B	108.6
C6—C7—H7A	108.9	C20—C22—H22B	108.6
C8—C7—H7A	108.9	H22A—C22—H22B	107.5
C6—C7—H7B	108.9	C24—C23—C22	113.5 (3)
C8—C7—H7B	108.9	C24—C23—H23A	108.9
H7A—C7—H7B	107.7	C22—C23—H23A	108.9
C14—C8—C7	110.5 (2)	C24—C23—H23B	108.9
C14—C8—C9	108.11 (19)	C22—C23—H23B	108.9
C7—C8—C9	112.1 (2)	H23A—C23—H23B	107.7
C14—C8—H8	108.7	C23—C24—C25	114.8 (3)
C7—C8—H8	108.7	C23—C24—H24A	108.6
C9—C8—H8	108.7	C25—C24—H24A	108.6
C8—C9—C11	109.6 (2)	C23—C24—H24B	108.6
C8—C9—C10	113.0 (2)	C25—C24—H24B	108.6
C11—C9—C10	113.8 (2)	H24A—C24—H24B	107.5
C8—C9—H9	106.6	C27—C25—C26	110.8 (3)
C11—C9—H9	106.6	C27—C25—C24	110.3 (3)
C10—C9—H9	106.6	C26—C25—C24	112.5 (3)
C19—C10—C1	108.5 (3)	C27—C25—H25	107.7
C19—C10—C9	110.1 (2)	C26—C25—H25	107.7
C1—C10—C9	110.2 (2)	C24—C25—H25	107.7
C19—C10—C5	113.7 (2)	C25—C26—H26A	109.5
C1—C10—C5	106.2 (2)	C25—C26—H26B	109.5
C9—C10—C5	108.0 (2)	H26A—C26—H26B	109.5
C12—C11—C9	112.2 (2)	C25—C26—H26C	109.5
C12—C11—H11A	109.2	H26A—C26—H26C	109.5
C9—C11—H11A	109.2	H26B—C26—H26C	109.5
C12—C11—H11B	109.2	C25—C27—H27A	109.5
C9—C11—H11B	109.2	C25—C27—H27B	109.5
H11A—C11—H11B	107.9	H27A—C27—H27B	109.5
C13—C12—C11	112.4 (2)	C25—C27—H27C	109.5
C13—C12—H12A	109.1	H27A—C27—H27C	109.5
C11—C12—H12A	109.1	H27B—C27—H27C	109.5
C13—C12—H12B	109.1	C3A—O3A—C3	119.3 (4)
C11—C12—H12B	109.1	O3B—C3A—O3A	122.4 (5)
H12A—C12—H12B	107.8	O3B—C3A—C3B	126.3 (5)
C12—C13—C18	110.1 (2)	O3A—C3A—C3B	111.2 (5)
C12—C13—C14	107.4 (2)	C3A—C3B—H3BA	109.5
C18—C13—C14	112.1 (2)	C3A—C3B—H3BB	109.5
C12—C13—C17	117.5 (2)	H3BA—C3B—H3BB	109.5
C18—C13—C17	109.5 (2)	C3A—C3B—H3BC	109.5
C14—C13—C17	99.84 (19)	H3BA—C3B—H3BC	109.5
C15—C14—C8	119.6 (2)	H3BB—C3B—H3BC	109.5
C15—C14—C13	104.3 (2)	C5—O5—H5A	109.5
C8—C14—C13	115.2 (2)	C6A—N6—C6	123.7 (3)
C15—C14—H14	105.5	C6A—N6—H6A	118.1
C8—C14—H14	105.5	C6—N6—H6A	118.1
C13—C14—H14	105.5	O6—C6A—N6	121.0 (3)
C14—C15—C16	103.4 (2)	O6—C6A—C6B	122.2 (3)
C14—C15—H15A	111.1	N6—C6A—C6B	116.9 (3)
C16—C15—H15A	111.1	C6A—C6B—H6BA	109.5
C14—C15—H15B	111.1	C6A—C6B—H6BB	109.5
C16—C15—H15B	111.1	H6BA—C6B—H6BB	109.5
H15A—C15—H15B	109.1	C6A—C6B—H6BC	109.5
C15—C16—C17	107.3 (2)	H6BA—C6B—H6BC	109.5
C15—C16—H16A	110.2	H6BB—C6B—H6BC	109.5
C17—C16—H16A	110.2		
			
C10—C1—C2—C3	−55.8 (4)	C11—C12—C13—C18	−69.1 (3)
C1—C2—C3—O3A	172.3 (3)	C11—C12—C13—C14	53.2 (3)
C1—C2—C3—C4	52.9 (4)	C11—C12—C13—C17	164.6 (2)
O3A—C3—C4—C5	−171.2 (2)	C7—C8—C14—C15	−52.3 (3)
C2—C3—C4—C5	−54.3 (4)	C9—C8—C14—C15	−175.3 (2)
C3—C4—C5—O5	−56.5 (3)	C7—C8—C14—C13	−177.8 (2)
C3—C4—C5—C6	−172.9 (2)	C9—C8—C14—C13	59.2 (3)
C3—C4—C5—C10	58.5 (3)	C12—C13—C14—C15	170.2 (2)
O5—C5—C6—N6	−172.7 (2)	C18—C13—C14—C15	−68.7 (3)
C4—C5—C6—N6	−55.2 (3)	C17—C13—C14—C15	47.1 (2)
C10—C5—C6—N6	72.6 (3)	C12—C13—C14—C8	−56.7 (3)
O5—C5—C6—C7	59.5 (3)	C18—C13—C14—C8	64.4 (3)
C4—C5—C6—C7	176.9 (2)	C17—C13—C14—C8	−179.8 (2)
C10—C5—C6—C7	−55.3 (3)	C8—C14—C15—C16	−166.5 (2)
N6—C6—C7—C8	−76.5 (3)	C13—C14—C15—C16	−36.0 (3)
C5—C6—C7—C8	51.8 (3)	C14—C15—C16—C17	10.6 (3)
C6—C7—C8—C14	−172.1 (2)	C15—C16—C17—C20	149.3 (2)
C6—C7—C8—C9	−51.4 (3)	C15—C16—C17—C13	18.2 (3)
C14—C8—C9—C11	−56.4 (3)	C12—C13—C17—C20	78.9 (3)
C7—C8—C9—C11	−178.5 (2)	C18—C13—C17—C20	−47.6 (3)
C14—C8—C9—C10	175.5 (2)	C14—C13—C17—C20	−165.5 (2)
C7—C8—C9—C10	53.4 (3)	C12—C13—C17—C16	−154.7 (2)
C2—C1—C10—C19	−66.1 (3)	C18—C13—C17—C16	78.8 (3)
C2—C1—C10—C9	173.3 (3)	C14—C13—C17—C16	−39.0 (2)
C2—C1—C10—C5	56.5 (3)	C16—C17—C20—C21	−178.0 (3)
C8—C9—C10—C19	70.0 (3)	C13—C17—C20—C21	−56.0 (4)
C11—C9—C10—C19	−55.8 (3)	C16—C17—C20—C22	56.9 (3)
C8—C9—C10—C1	−170.2 (2)	C13—C17—C20—C22	179.0 (2)
C11—C9—C10—C1	63.9 (3)	C21—C20—C22—C23	57.8 (4)
C8—C9—C10—C5	−54.6 (3)	C17—C20—C22—C23	−176.2 (2)
C11—C9—C10—C5	179.5 (2)	C20—C22—C23—C24	167.7 (3)
O5—C5—C10—C19	178.1 (2)	C22—C23—C24—C25	−178.6 (3)
C4—C5—C10—C19	61.3 (3)	C23—C24—C25—C27	167.7 (4)
C6—C5—C10—C19	−66.4 (3)	C23—C24—C25—C26	−68.1 (4)
O5—C5—C10—C1	58.8 (3)	C2—C3—O3A—C3A	152.4 (5)
C4—C5—C10—C1	−58.0 (3)	C4—C3—O3A—C3A	−87.0 (5)
C6—C5—C10—C1	174.3 (2)	C3—O3A—C3A—O3B	7.4 (10)
O5—C5—C10—C9	−59.4 (3)	C3—O3A—C3A—C3B	−175.4 (5)
C4—C5—C10—C9	−176.2 (2)	C7—C6—N6—C6A	−98.2 (3)
C6—C5—C10—C9	56.1 (3)	C5—C6—N6—C6A	135.0 (3)
C8—C9—C11—C12	57.1 (3)	C6—N6—C6A—O6	0.7 (5)
C10—C9—C11—C12	−175.3 (2)	C6—N6—C6A—C6B	180.0 (3)
C9—C11—C12—C13	−56.5 (3)	C19—C10—C13—C18	6.3 (2)

### Hydrogen-bond geometry (Å, °)

**Table:**

*D*—H···*A*	*D*—H	H···*A*	*D*···*A*	*D*—H···*A*
O5—H5A···O6^i^	0.82	1.99	2.804 (3)	172

Symmetry codes: (i) −*x*+2, *y*, −*z*+1.
